# Use of Disposable Clear Plastic Elastic Band-Rimmed Bag to Limit Infectious Aerosol during Airway Instrumentation

**DOI:** 10.7759/cureus.10896

**Published:** 2020-10-11

**Authors:** Cynthia M Wong, Apolonia E Abramowicz

**Affiliations:** 1 Anesthesiology, Westchester Medical Center/New York Medical College, Valhalla, USA

**Keywords:** coronavirus disease, covid 19, covid-19 (corona-virus disease), sars-cov-2, airway extubation, health care worker safety, elastic band rimmed bag, banded bag, negative pressure, barrier enclosure

## Abstract

The coronavirus disease 2019 (COVID-19) pandemic has highlighted the need for appropriate protective measures for health care providers, particularly for those involved in aerosol-generating procedures. We report the use of the banded bag for extubation to contain infectious aerosols. The banded bag is a clear and disposable shower-cap style image intensifier cover which is commonly used as a sterile cover for mobile X-ray systems. With the addition of a filtered suction, safe air exchange rates can be obtained. We anticipate that the banded bag, which is economical, convenient, and highly practical, can be used as a safety-enhancing device for COVID-19 extubations.

## Introduction

The severe acute respiratory syndrome coronavirus 2 (SARS-CoV-2) virus responsible for coronavirus disease 2019 (COVID-19) has caused a global pandemic due to the infectivity of the virus particles. Those who are involved in aerosol-generating procedures are at particularly high risk due to the potential for exposure to infectious respiratory secretions. Current recommendations for the management of COVID-19 suspected or confirmed patients include personal protective equipment (PPE) and negative-pressure rooms [1]. However, the virus may still pose an infective risk in airborne particles for hours and on surfaces for days [2]. Both intubation and extubation of patients infected with COVID-19 are considered aerosol-generating procedures and therefore carry risk to the proceduralist. Of these two, extubation is a much less controlled maneuver due to the risk of coughing. One group described a “mask over tube” technique for extubation, where a face mask air-cushion seal is maintained as the endotracheal tube is removed sideways to minimize staff exposure [3]. While aerosol and contact precautions, including PPE usage, are mandatory during airway management, some practitioners have taken to utilizing forms of patient barriers for added protection.

Simulations have demonstrated that without the use of a barrier, simulated droplets and aerosols can be found on the gown, gloves, face mask, eye shield, hair, neck, ears, and shoes of the intubater [4-6]. Some have utilized a plastic intubation box while others have utilized a clear plastic drape over a patient’s head during intubation and extubation in order to prevent respiratory droplets and aerosols from spreading [4,5,7]. These barriers have shown reduced contamination to the area around the patient in simulation [4,5]. However, the drape is sometimes difficult to position and its removal creates the potential for contact with fomites on its underside. Meanwhile, plastic boxes have less maneuverability, may expose other staff to infection, and require decontamination for storage and reuse [8]. Recently, the Food and Drug Administration has made recommendations that passive protective barrier enclosures should not be used without negative pressure; they have stated that “the known and potential benefits for emergency use of these devices, when used as authorized, continue to outweigh the known and potential risks and do not present public health or safety concerns at this time” [9]. Other designs have since incorporated the use of negative pressure to increase air exchange rates, however, these designs are encumbered by a frame, which reduces the designs’ versatility [10,11].

## Technical report

**Figure 1:**
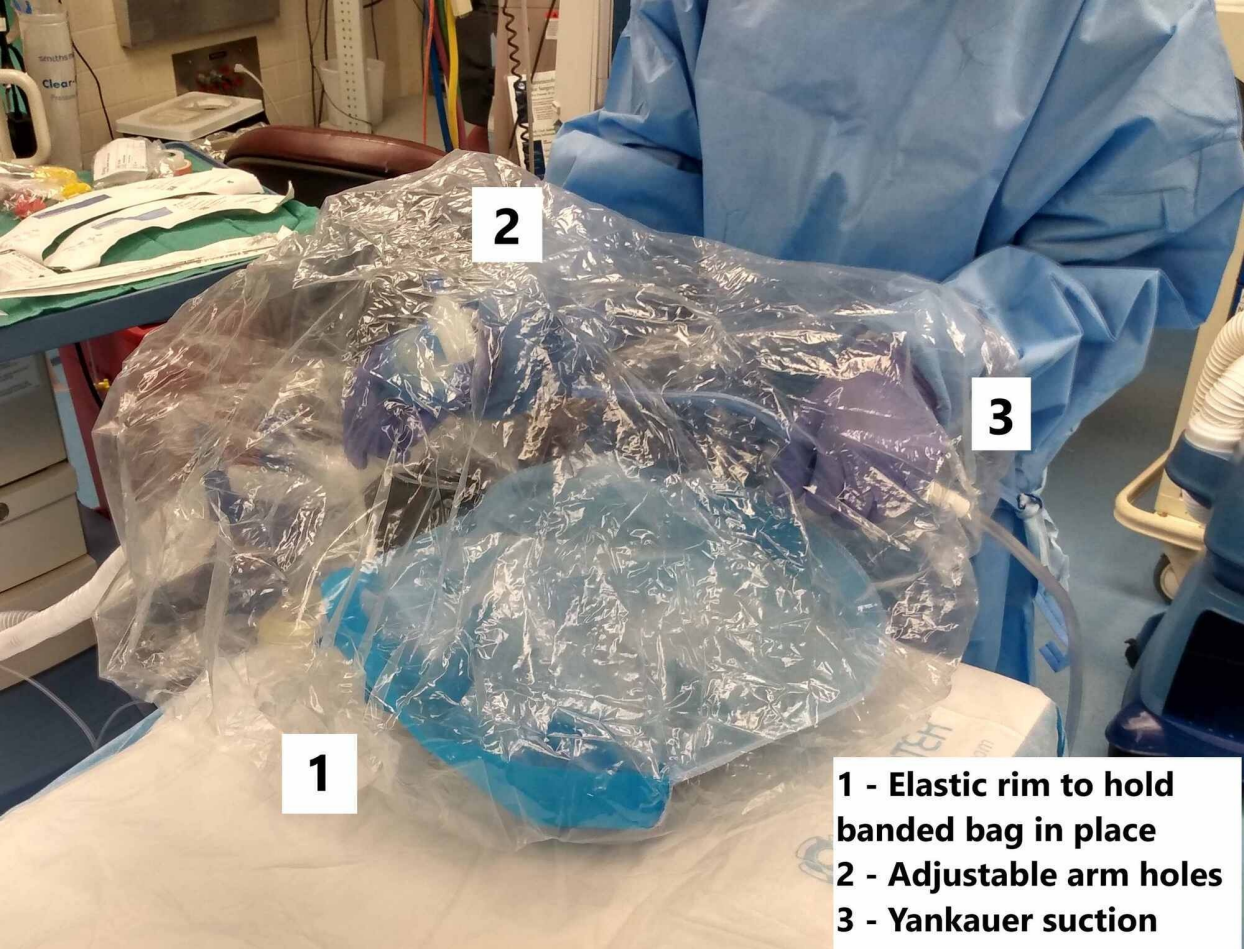
Demonstration of the banded bag The elastic rim surrounds the pillow, and in practice, the patient’s head. Customizable armholes are cut into the plastic to allow for ease of access while minimizing exposure to the outside environment. A filtered suction is included within the design for negative pressure air exchange rates.

We utilize the clear, disposable shower-cap style sterile image intensifier cover, which can be used with fixed mono or bi-plane X-ray systems (SNAP KAP™ [Advance Medical Designs Inc, Marietta, GA] seamless latex-free equipment cover with 66 cm depth) during the extubation of infective or potentially infective patients (Figure 1). The elastic rim encompasses the pillow under a patient’s head. While the patient is still intubated, arm holes are cut into the bag. Placement for the arm holes can be made according to operator size and preference. A Yankauer suction tip (Cardinal Health, Waukegan, IL) with a high-efficiency particulate air (HEPA) filter is introduced to the system via another hole and placed near the patient’s airway. This suction should be attached to a high-volume extraction suction system (Stryker Neptune™, Kalamazoo, MI). The circuit within the bag can be easily attached to a face mask placed within the bag in anticipation of extubation; this should minimize the degree of seal disruption. After tracheal extubation, the endotracheal tube and the mask can be wrapped in the surrounding bag and easily disposed of without risk of contaminating the outside environment.

## Discussion

Technological principles and viability

With the addition of a suction with a HEPA filter to the elastic band-rimmed bag, a negative pressure of -200 mmHg can be generated for a flow of 80.2 liters per minute of airflow. After the addition of the vacuum regulator, collection canister, and standard suction tubing, the airflow drops to 47.6 Lmin-1 [12]. Within other COVID-19 airway management enclosures, air exchange rates of 35-37 Lmin-1 have been calculated, while the high-volume extraction suction was calculated at 118 Lmin-1, with the decreased air exchange rate compared to previously published data likely due to the addition of the viral filter [13]. This corresponds to an air change per hour of 10.6 when using the standard filtered suction and 34.8 with the high volume extraction suction. The Center for Disease Control and Prevention recommends 15 air changes per hour and 12 for negative pressure rooms, so the use of the high volume extraction suction with the banded bag would be required to achieve safe air changes per hour [14].

Advantages of the banded bag

The elastic band-rimmed bags are readily available, inexpensive, and practical. The banded bag has an elastic rim which allows droplets and aerosols to be fully contained within the dome cover and minimizes contamination to the bed underneath; adding a filtered suction makes evacuation of aerosol contamination even more effective. The bags are highly portable and can be utilized on a wide variety of surfaces, including standard operating tables and all forms of hospital bed and stretcher. This is in contrast to other devices, which can only fit beds of a certain width, or have bulky frames that may not suit a wide variety of situations. The choice of arm hole placement allows providers optimization for specific extubation conditions when utilizing the device. Unlike some box designs, the arm holes of the banded bag are unlikely to damage PPE [15]. The arm holes can also be cut to match an operator’s forearm size to minimize the escape of air particles. The elasticated rim structure helps prevent accidental fomite contact with the interior during removal and disposal of the bag. It also has the added benefit of containing the recently extubated endotracheal tube, which never has to be exposed to the outside environment. Clean-up can be performed more efficiently and safely when compared to drapes which have the potential to cause contamination during their clean-up, and frames, which are unwieldy and if reused, can be cumbersome to clean. Those devices that have suctioning within the frame pose the issue of difficulty cleaning the interior of the frame [10].

Limitations of the banded bag

The banded bag is not without limitations. It may be restricting when attempting intubation, particularly if long intubating aids are used. The wrinkled plastic can also obscure the intubator’s vision, making direct laryngoscopy more technically challenging. Given this limitation, the banded bag is better suited to extubation, which requires less space within the dome. Suction availability is a consideration, although when the banded bag is used in an operating room or ICU setting, suction should be readily available. The forearms of the operator will be covered in infectious fomites, so caution should be exercised when removing the arms and doffing. There is the potential of escape of infectious aerosols after removal of the arms, however, this risk is reduced with effective suction and if the holes are covered after arm removal with tape or a clear plastic adhesive film. Considering the need for training and practice with using the banded bag, it is essential that there is adequate buy-in from practitioners. It may be possible that airborne particle contamination when using an aerosol barrier enclosure is increased compared to no device use; this has been hypothesized to be due to the Bernoulli principle causing air particles to escape through arm holes [16]. It is unknown if this effect is also present with more form-fitting arm holes. In the event of airway compromise during extubation, there should be a low threshold for abandoning the use of the banded bag if it puts securing the airway at risk.

## Conclusions

As we face the possibility of a second wave of an infectious outbreak in the near future, we must be innovative and resourceful to keep healthcare workers safe. We anticipate that the banded bag, which is economical, convenient, and highly practical, can be used as a safety-enhancing device for COVID-19 extubations. However, further studies should be performed to compare methods of aerosol containment, and to measure air exchange rates, air turbulence, and flow rates in greater quantitative detail. These studies of safety, usability, and efficacy should be conducted before regular use can be recommended.
